# Efficacy of local infiltration analgesia with ropivacaine for postoperative pain management in cervical laminoplasty: a retrospective study

**DOI:** 10.1038/s41598-020-61229-2

**Published:** 2020-03-06

**Authors:** Kunpeng Li, Hao Li, Dawei Luo, Hongyong Feng, Changbin Ji, Keshi Yang, Jinlong Liu, Honglei Zhang, Hui Xu

**Affiliations:** Department of Orthopaedics, Liaocheng People’s Hospital, Liaocheng, China

**Keywords:** Clinical trials, Pain

## Abstract

Poor postoperative pain control impairs patient recovery and lengthens the duration of hospitalization after various surgeries. Local infiltration analgesia(LIA) has become an effective method for managing postoperative pain. This study aimed to investigate the efficacy of LIA with ropivacaine for postoperative pain control after cervical laminoplasty. In total, 68 patients undergoing cervical laminoplasty were included for retrospective review and divided into ropivacaine and control groups. The visual analogue scale (VAS) score, postoperative analgesic consumption, operative duration, intraoperative blood loss volume, incision length, hospitalization duration and incidence of complications were analyzed. In the ropivacaine group, the VAS score was 3.2 ± 1.4 at 4 hours postoperatively, which was lower than that of the control group(4.0 ± 1.4, P = 0.024). At 8, 12 and 24 hours after surgery, a significant difference was detected in the VAS score between the two groups(P ≤ 0.015). Sufentanil consumption was less in the ropivacaine group than in the control group in the first 4 hours postoperatively (25.6 ± 6.3 µg vs 32.2 ± 6.8 µg, P < 0.001), and similar results were observed in the first 8, 12, 24, 48 and 72 hours postoperatively(P < 0.001). Fewer patients required rescue analgesia in the ropivacaine group(8/33 vs 18/35 at 4–8 hours, P = 0.021; 9/33 vs 21/35 at 8–12 hours, P = 0.007). The hospitalization duration and time to ambulation were shorter in the ropivacaine group(8.5 ± 1.4 vs 9.6 ± 1.6 for postoperative duration, P = 0.002; 2.9 ± 0.7 vs 3.5 ± 0.8 for time to ambulation, P = 0.001). The incidence of nausea and vomiting was lower in the ropivacaine group than in the control group(30.3% vs 54.3%, P = 0.046). In conclusion, LIA with ropivacaine could effectively reduce postoperative pain, and postoperative analgesic consumption, and promote recovery after cervical laminoplasty.

## Introduction

Cervical laminoplasty has been used widely and achieved satisfactory outcomes in treating multilevel cervical lesions, including cervical spondylotic myelopathy(CSM), congenital cervical stenosis(CCS), and ossification of the posterior longitudinal ligament(OPLL)^1–3^. However, surgical treatment often results in serious pain, which can impair patient recovery and lengthen the hospitalization duration.

Recently, local infiltration analgesia (LIA) has emerged as a good choice for postoperative analgesia due to its simplicity and low-cost^4,5^. LIA has been used in various surgeries with favorable outcomes and without major side effects^6^. The benefits of LIA in spinal surgery are also well known; however, its use has been limited to lumbar decompression and discectomy, and spinal fusion surgery^7,8^. Few studies have focused on the effect of LIA on postoperative pain control in cervical laminoplasty.

Therefore, we conducted this clinical study to retrospectively evaluate the efficacy of LIA with ropivacaine for managing postoperative pain after cervical laminoplasty.

## Methods and Materials

### Patient population

This was a retrospective study conducted in the Department of Spinal Surgery, Liaocheng People’s Hospital, between January 2014 and December 2016. The inclusion criteria were as follows: age 18–75 years; primary diagnosis of CSM, CCS, or OPLL, involvement of 3 or more segments; and treatment with expansive open-door cervical laminoplasty. The exclusion citeria were American Society of Anesthesiologists physical status III or higher, history of cervical surgery, and the presence of myelopathy caused by trauma, tumors or infections.

All patients who underwent posterior cervical laminoplasty from January 2014 to December 2016 were reviewed. Only 68 patients who met the inclusion criteria and provided written informed consent were included in this trial. The enrolled patients were divided into two groups: 33 patients which received LIA with ropivacaine comprised the ropivacaine group, and 35 patients who did not receive LIA comprised the control group The study was approved by the Ethics Committee of Liaocheng People’s Hospital, and written informed consent was provided by every patient before enrollment. All procedures in this study were performed according to the relevant guidelines and regulations.

Previous literature has reported that a difference of 1.0 to 1.3 points on the visual analogue scale (VAS) is clinically important^9^. Elder^10^ showed a standard deviation of 1.97 on the VAS following cervical laminoplasty. Additionally, we reviewed several similar reports and found that the number of the treated subjects was approximately 30^10–12^. Therefore, we determined that a total of 30 patients per group was required to detect a 1.5-point difference on the VAS (power = 80%, p = 0.05). The power was 0.815 according to data mentioned above(n = 30, σ = 2.0, δ = 1.5, α = 0.05). The sample size and power analysis were performed using the *Power and Sample Size Program(Power and Sample Size Calculation version 3.1.6, Copyright © 1997–2009 by William D. Dupont and Walton D. Plummer, Jr)*.

### Surgical procedure

The surgical procedure was performed as described in a previous report by Li^13^. All patients received general anesthesia with 0.1% propofol, dexmedetomidine, fentanyl, remifentanil and cisatracurium. The patients were positioned in prone with slight flexion of the neck in a Mayfield head fixator. A standard posterior midline excision was performed and the lamina were exposed to medial facet joints from C3 to C7. A hinge was made on the side with fewer symptoms by removing the dorsal cortex and cancellous bone, while the lamina along the medial margin of the facet joints was completely removed on the open side. After elevating the opening lamina approximately 1 cm, a titanium plate was placed on each segment. Two screws were implanted to fix the plate tightly to the lamina and lateral mass. Electrocardiography, blood pressure, pulse oximetry, and arterial blood gas were routinely monitored.

No preemptively scheduled analgesic regimen was employed.

### LIA

Before the incision line was closed after decompression and fixation, 10 ml of ropivacaine (concentration: 0.75%) was administered over the incision line into paravertebral muscles, and the subcutaneous, and cutaneous tissue along the wound edge. After surgery, the patient was placed in the supine position, and was extubated successfully on the table. Once the patient was awake and responding to verbal commands, he or she was transferred to the postanesthesia care unit, and then to the spine ward for further monitoring and recovery care.

### Postoperative management

Every patient received patient-controlled analgesia (PCA) with 0.8 µg/ml sufentanil for 72 hours postoperatively. The PCA pump was set so that one pump press delivered a 2-ml bolus with no continuous background infusion. The sufentanil was given at a bolus of 2 ml (1.6 µg) with a 5-minute lockout time, and the maximum dosage was 12.8 µg per hour. When a patient indicated a VAS score ≥5, flurbiprofen axetil was given as a rescue analgesic.

Prophylactic antibiotics were administered for 24 hours after surgery. All patients were encouraged to start out-of-bed activity with a cervical brace within one week after surgery. Mechanical thromboprophylaxis was given to every patient to prevent phlebothrombosis in both legs. Clinical and radiological assessments were performed in the orthopedic outpatient clinic every three months after discharge from the hospital.

### Observed indexes

The postoperative VAS score and sufentanil consumption were recorded at 4, 8, 12, 24, 48 and 72 hours after surgery to evaluate pain severity. The number of patients who required rescue analgesia (flurbiprofen axetil) was also recorded postoperatively.

The operative duration, intraoperative blood loss volume and incision length were quantified to evaluate the surgical trauma. The recovery time including the length of total and postoperative hospital duration and the time to ambulation was recorded.

The incidence of complications, including postoperative nausea and vomiting(PONV) and wound infection, was also analyzed in this study.

### Statistical analyses

Data analyses were performed with the SPSS 17.0 statistical package (SPSS, Chicago, U.S.A.). In this study, continuous data, including VAS, sufentanil consumption, operative indexes and recovery time, are presented as the mean±standard deviation and were analyzed with the two-sample t-test and ANOVA. The chi-squared test was used to analyze categorical data, such as the number of patients requiring rescue analgesia and the incidence of complications. All reported p values were from 2-sided tests. A P value lower than 0.05 was regarded as statistically significant.

## Results

In this study, 68 patients undergoing posterior expansive open-door cervical laminoplasty were included, with 33 in the ropivacaine group and 35 in the control group. The patients’ demographics information and basic characteristics, including age, sex, weight, height, BMI and fracture level are shown in Table 1 and there were no significant differences between the two groups.

**Table 1:** Characteristics of the patients in both groups.

Parameter	Ropivacaine group	Control group	P value
Number of patients	33	35	
Age(year)	60.0 ± 6.6 (49–70)	59.2 ± 7.4 (47–75)	0.62
Sex, male/female	25/10	22/11	0.68
Weight(kg)	68.2 ± 8.0	67.2 ± 7.8	0.60
Height(cm)	169 ± 6.9	169.7 ± 6.9	0.67
BMI(kg/m^2^)	23.8 ± 1.8	23.3 ± 1.9	0.23
Diagnosis			0.79
CSM	11	13	
OPLL	8	10	
CSS	14	12	

CCS = cervical canal stenosis, CSM = cervical spondylotic myelopathy, OPLL = ossification of the posterior longitudinal ligament.

### Operative indexes

The operative duration was slightly longer in the ropivacaine group (121 ± 24 min) than in the control group (117 ± 19 min), but no significant difference was observed (P = 0.46). Similar results were observed between the two groups for the intraoperative blood loss volume and incision length (295.2 ± 75.1 ml vs 310.6 ± 80.2 ml, P = 0.42; 14.6 ± 1.2 cm vs 14.2 ± 1.1 cm, P = 0.25, ropivacaine vs control group, respectively) (Table 2).

**Table 2:** Operative indexes for both groups.

Operative index	Ropivacaine group	Control group	P value
Operative duration (min)	121 ± 24	117 ± 19	0.46
Intraoperative blood loss volume (ml)	295.2 ± 75.1	310.6 ± 80.2	0.42
Incision length (cm)	14.6 ± 1.2	14.2 ± 1.1	0.25

### Recovery time

The average total hospitalization duration was significantly shorter in the ropivacaine group(10.6 ± 1.5 days) than in the control group (11.7 ± 1.6 days, P = 0.006). Significant differences were found in the postoperative hospitalization and the time to ambulation between the ropivacaine and control groups (P = 0.002 and P = 0.001, respectively) (Table 3).

**Table 3:** Recovery time for the patients in both groups.

Recovery time	Ropivacaine group	Control group	P value
Total hospitalization duration (days)	10.6 ± 1.5	11.7 ± 1.6	0.006
Postoperative hospitalization duration(days)	8.5 ± 1.4	9.6 ± 1.6	0.002
Time to ambulation(days)	2.9 ± 0.7	3.5 ± 0.8	0.001

### Evaluation of pain severity

**Figure 1:**
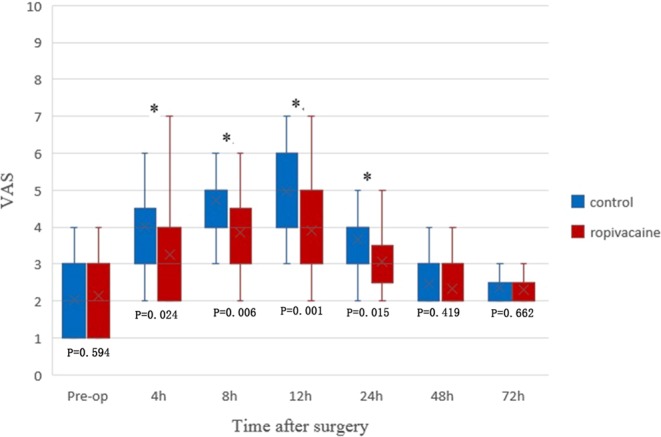
Boxplot showing VAS scores over the first 72 h postoperatively for the ropivacaine and control groups. The boxes indicate the interquartile range, the crosses within the boxes indicate the median, and the whiskers indicate the range. The asterisks indicate significance (P < 0.05); h = hours postoperatively.

**Figure 2:**
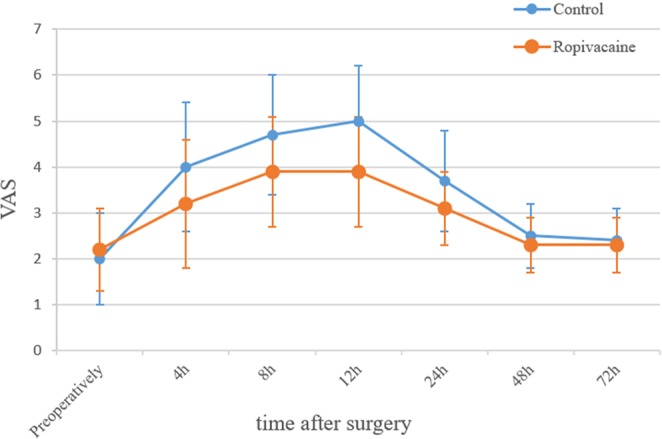
Line graph showing the change in VAS scores over time. The mean VAS score and error bar are plotted for each time point for the patients in the ropivacaine group and the control group. h = hours postoperatively.

The average VAS score 4 hours postoperatively was lower in the ropivacaine group(3.2 ± 1.4 points) than in the control group(4.0 ± 1.4 points, P = 0.024). At 8, 12 and 24 hours after surgery, there was a significant difference in the VAS score between the two groups(P ≤ 0.015). However, no significant difference was found at 48 hours and 72 hours postoperatively(P = 0.42 and P = 0.66, respectively) (Fig. 1). The change in VAS scores over time in the first 72 hours postoperatively was shown in Fig. 2.

### Analgesic consumption

**Figure 3:**
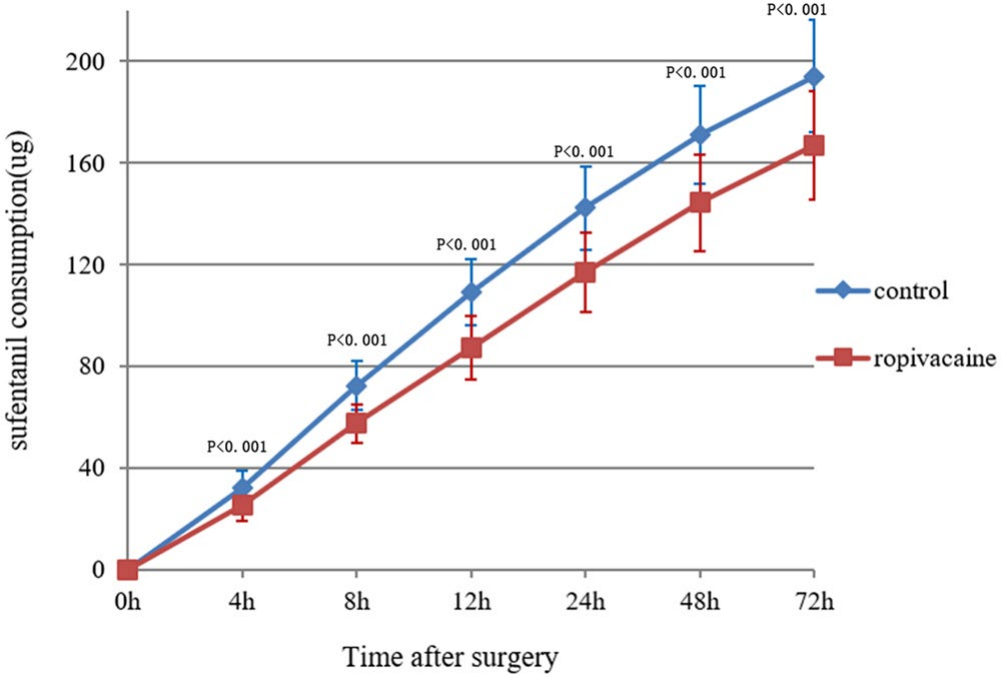
Line graph showing the cumulative sufentanil consumption over the first 72 h postoperatively for the ropivacaine and control groups. Statistical significance was found at each time point over the first 72 h after surgery; h = hours postoperatively.

The sufentanil consumption in the ropivacaine group was less than the control group in the first 4 hours postoperatively (25.6 ± 6.3 µg vs 32.2 ± 6.8 µg, P < 0.001). Similar results were found regarding the cumulative sufentanil consumption in the first 8, 12, 24, 48 and 72 hours after surgery(P < 0.001) (Fig. 3).

**Figure 4:**
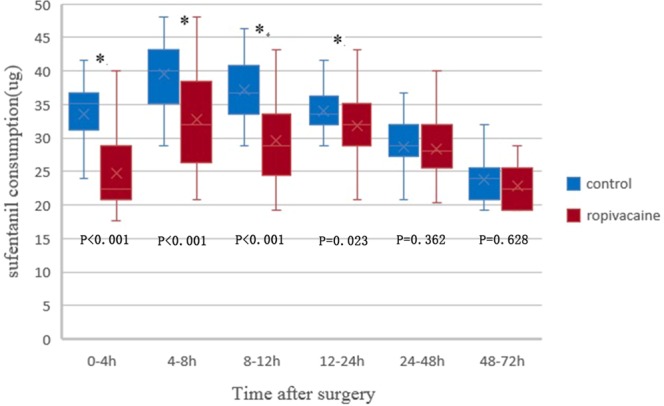
Boxplot showing sufentanil consumption at 0–4 h,4–8 h, 8–12 h, 12–24 h, 24–48 h and 48–72 h postoperatively for the ropivacaine and control groups. The boxes indicate the interquartile range, the crosses within the boxes indicate the median, and the whiskers indicate the range. The asterisks indicate significance (P < 0.05); h = hours postoperatively.

**Figure 5:**
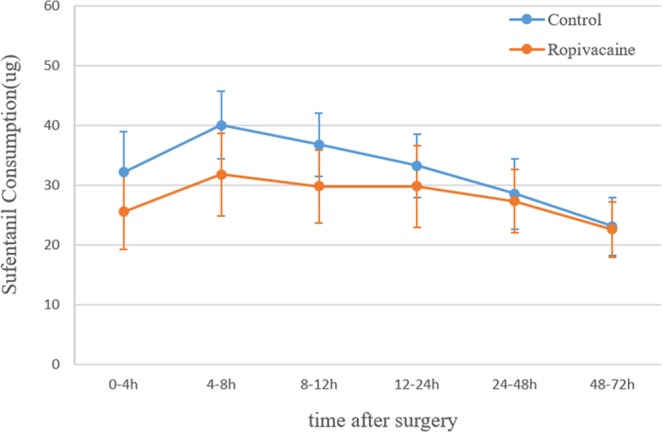
Line graph showing the change in sufentanil consumption over time. The mean sufentanil consumption and error bar are plotted for each period for the patients in the ropivacaine group and the control group. h = hours postoperatively.

In the ropivacaine group, the sufentanil consumption was 31.8 ± 6.9 µg from 4–8 hours postoperatively, which was less than that in the control group (40.1 ± 5.7 µg, P < 0.001). Similar results were found from 8–12 hours and 12–24 hours postoperatively (P < 0.001 and P = 0.023). However, no significant difference was observed from 24–48 hours, or 48–72 hours between the two groups (P = 0.36 and P = 0.63) (Fig. 4). The change in sufentanil consumption over time in the first 72 hours postoperatively was shown in Fig. 5.

Five patients in the ropivacaine group and 9 in the control group required the administration of flurbiprofen axetil in the first 4 hours after surgery, with no significant difference between the groups (P = 0.28). However, there was a significant difference in the number of patients requiring flurbiprofen axetil in the 4–8 hours and 8–12 hours after surgery(8/33 vs 18/35, P = 0.021; 9/33 vs 21/35, P = 0.007) (Table 4).

**Table 4:** Administration of flurbiprofen axetil in both groups.

Period	Ropivacaine group	Control group	P value
First 4 hours	5/33	9/35	0.28
4–8 hours	8/33	18/35	0.021
8–12 hours	9/33	21/35	0.007

### Complications

No case of clinical deterioration, permanent morbidity or mortality occurred in this study. In terms of the incidence of PONV, a significant difference was observed between the two groups (30.3% vs 54.3%, ropivacaine vs control group, P = 0.046). There was one case of wound infection in each group, and in both cases, the patient recovered after routine antibiotic treatment and dressing changes (Table 5).

**Table 5:** Incidence of complications in both groups.

Complication	Ropivacaine group	Control group	P value
PONV	10/33	19/35	0.046
Wound infection	1/33	1/35	>0.99

## Discussion

In the current study, we adopted LIA with 0.75% ropivacaine in posterior cervical laminoplasty to assess its efficacy in postoperative pain management. The results show significantly better outcomes for most parameters in the ropivacaine group than in the control group. LIA with ropivacaine could reduce pain severity and the consumption of opioid drugs via PCA after surgery and decrease the number of patients who required rescue analgesia. The data further indicated a lower incidence of PONV and a shorter hospitalization duration in the ropivacaine group.

LIA has been used in many surgeries as a pain control technique, and a series of reports has documented the use of this technique and its satisfactory outcomes in orthopedic, gynecological and abdominal surgeries. Sun and colleagues^14^ performed wound ropivacaine infiltration in patients undergoing open hepatectomy and found that it could reduce pain severity and surgical stress response, and could improve postoperative recovery. O’Neill^15^ reported that continuous ropivacaine infiltration in the first 48 hours achieved better analgesia, with fewer side effects and a shorter hospitalization duration after cesarean delivery. Koehler^16^ conducted a randomized controlled trial and concluded that surgical-site injection alleviated postoperative pain and reduced opioid utilization on the first day postoperatively without major adverse events in femoral fracture patients undergoing surgical treatment.

PCA is one technique that is commonly used to control postoperative pain after major spinal surgery^17,18^. Since Mullen and Cook first reported the use of LIA in spine surgery in 1979^19^, several authors have adopted LIA for lumbar spinal surgery and found that it could reduce the severity of postoperative pain and decrease the consumption of opioid analgesics. In the current study, we assessed the severity of postoperative pain using the VAS score and the consumption of sufentanil after posterior cervical surgery. The VAS scores in the first 24 hours were obviously lower in the patients who received LIA than the control patients, and the total opioid use via PCA within 72 hours was also less in the LIA patients than in the control patients. These findings are similar to those of previous studies^14,20,21^. Furthermore, we calculated the periodic consumption of sufentanil in the first 72 hours after surgery and found that the ropivacaine group used less opioid analgesics than the control group at each follow-up point in the first 24 hours postoperatively.

Elder^10^ adopted continuous anesthesia with bupivacaine via an elastomeric pump during posterior cervical spinal surgery, and found that continuous anesthetic infusion could achieve better outcomes in managing postoperative pain with lower pain scores and less opioid use. That author also considered that a single dose of bupivacaine could not provide sustained effects in the management of postoperative pain, because of the inherently short effective period of bupivacaine allowed by its bioavailability. This is not consistent with our finding that LIA with ropivacaine could provide pain relief over the first 24 hours after surgery. This difference may be attributed to the longer duration of ropivacaine administration during the LIA procedure compared with that of bupivacaine. Previous studies^22,23^ have confirmed that as a propyl analog of bupivacaine, ropivacaine has a longer duration of action and is much safer than bupivacaine in terms of the cardiotoxicity profile.

In the current study, we recorded several operative indexes including the operative duration, intraoperative blood loss volume, and incision length, to assess the surgical trauma caused by posterior cervical laminoplasty. These indexes were similar in both groups and no significant differences were detected, which suggests that the surgical trauma to the patients was comparable. This finding indicates that the surgery caused postoperative pain of a similar severity in the two groups.

Effective pain management is now recognized as one of the three fundamental aspects of enhanced recovery after surgery^24^. As a potentially effective fast-track method, the role of postoperative pain control has not been well established in posterior cervical laminoplasty. Therefore, we investigated the role of LIA with ropivacaine in the recovery of patients undergoing treatment with cervical laminoplasty. In our study, the results demonstrate that the total hospitalization duration of the ropivacaine group was shorter than that of the control group. Rao reported that the postoperative hospital stay was a better indicator of patient recovery^11^. The use of LIA with ropivacaine could also shorten the postoperative hospitalization duration, which suggests that the use of LIA with ropivacaine could effectively enhance the recovery of patients after cervical laminoplasty. Another indicator that could reflect patient recovery is the time to ambulation. The time to ambulation in the ropivacaine group was shorter than that in the control group, indicating that the wound infiltration with ropivacaine could decrease postoperative pain and promote the earlier initiation of out-of-bed activity. Thus, the use of LIA with ropivacaine for anesthesia could be effective in promoting patient recovery after surgery. These results are consistent with other previous reports^11,25^.

PONV is a very common complication following opioid-based intravenous PCA^26^. PONV can cause dehydration, electrolyte imbalance, postoperative bleeding, wound dehiscence, and pulmonary aspiration, and further aggravate patient discomfort. In a randomized controlled trial, Li^27^ found that wound ropivacaine infiltration could decrease the incidence of PONV in patients receiving intravenous morphine for analgesia after lumbar fusion surgery. These results were confirmed by our research. Fewer patients who received the LIA with ropivacaine experienced PONV than patients in the control group, which may be due to the less consumption of sufentanil by those who received LIA with ropivacaine.

There are some limitations to this study that impair the ability to evaluate the effect of LIA with ropivacaine on postoperative pain management. First, this trial was retrospective, not randomized and not blinded, and was performed at a single center. Second, this study included a small number of patients. Prospective, randomized controlled studies, enrolling more patients and spanning multiple centers, are needed to further evaluate the efficacy of ropivacaine for managing postoperative pain after cervical laminoplasty.

## Conclusion

In conclusion, LIA with ropivacaine could effectively reduce postoperative pain severity and postoperative analgesic consumption after cervical laminoplasty. Moreover, it could promote recovery after surgery.
